# Measuring and Modeling Oxygen Transport and Consumption in 3D Hydrogels Containing Chondrocytes and Stem Cells of Different Tissue Origins

**DOI:** 10.3389/fbioe.2021.591126

**Published:** 2021-05-25

**Authors:** Simon F. Carroll, Conor T. Buckley, Daniel J. Kelly

**Affiliations:** ^1^Trinity Centre for Biomedical Engineering, Trinity Biomedical Sciences Institute, Trinity College Dublin, Dublin, Ireland; ^2^Department of Mechanical, Manufacturing and Biomedical Engineering, School of Engineering, Trinity College Dublin, Dublin, Ireland; ^3^Department of Anatomy, Royal College of Surgeons in Ireland, Dublin, Ireland; ^4^Advanced Materials and BioEngineering Research (AMBER) Centre, Trinity College Dublin, Dublin, Ireland

**Keywords:** tissue engineering, oxygen consumption, cellular metabolism, chondrogenesis, stem cell differentiation, cartilage

## Abstract

Understanding how the local cellular environment influences cell metabolism, phenotype and matrix synthesis is crucial to engineering functional tissue grafts of a clinically relevant scale. The objective of this study was to investigate how the local oxygen environment within engineered cartilaginous tissues is influenced by factors such as cell source, environmental oxygen tension and the cell seeding density. Furthermore, the subsequent impact of such factors on both the cellular oxygen consumption rate and cartilage matrix synthesis were also examined. Bone marrow derived stem cells (BMSCs), infrapatellar fat pad derived stem cells (FPSCs) and chondrocytes (CCs) were seeded into agarose hydrogels and stimulated with transforming growth factor-β3 (TGF- β3). The local oxygen concentration was measured within the center of the constructs, and numerical modeling was employed to predict oxygen gradients and the average oxygen consumption rate within the engineered tissues. The cellular oxygen consumption rate of hydrogel encapsulated CCs remained relatively unchanged with time in culture. In contrast, stem cells were found to possess a relatively high initial oxygen consumption rate, but adopted a less oxidative, more chondrocyte-like oxygen consumption profile following chondrogenic differentiation, resulting in net increases in engineered tissue oxygenation. Furthermore, a greater reduction in oxygen uptake was observed when the oxygen concentration of the external cell culture environment was reduced. In general, cartilage matrix deposition was found to be maximal in regions of low oxygen, but collagen synthesis was inhibited in very low (less than 2%) oxygen regions. These findings suggest that promoting an oxygen consumption profile similar to that of chondrocytes might be considered a key determinant to the success of stem cell-based cartilage tissue engineering strategies.

## Introduction

The efficacy of stem cell based cartilage tissue engineering strategies is typically evaluated based on changes in the expression of chondrogenic matrix related genes, as well as the resulting biochemical composition and biomechanical function of the graft (Kafienah and Sims, 2004; Cernanec et al., 2007; Fehrer et al., 2007; Buckley et al., 2010; Meyer et al., 2010; Farrell et al., 2012, 2014; Thorpe et al., 2012; Carroll et al., 2014). However, other changes in phenotype, such as cellular metabolic activity, may play an equally important role in determining whether a stem cell can differentiate to fulfill a tissue specific function. In the case of cartilage tissue engineering strategies, it would seem reasonable to assume that chondrogenically primed mesenchymal stem cells (MSCs) would need to adopt oxygen and nutrient consumption rates approaching that of chondrocytes in order to thrive following implantation into an articular cartilage defect.

Chondrocytes are known to reside in an avascular, low oxygen *in vivo* environment, which ranges from around 6% in the superficial zone to as low as 1% in the deep zone (Zhou et al., 2004; Fermor et al., 2007). MSCs, however, typically reside in tissues such as bone marrow, which has been shown to possess oxygen levels in the range of 4–7% *in vivo* (Grant and Smith, 1963; Kofoed et al., 1985; Harrison et al., 2002), and due to their perivascular niche (as reviewed by Caplan, 2007; da Silva Meirelles et al., 2008), are likely to experience oxygen levels at least at the higher end of this range. This would suggest that undifferentiated MSCs possess a different oxygen consumption rate to that of differentiated chondrocytes. Therefore, determining if MSCs used to engineer cartilage grafts adopt a metabolic profile similar to chondrocytes might be considered a key determinant of the success of stem cell-based cartilage tissue engineering strategies. Furthermore, given that MSC phenotype and its biosynthetic activity is influenced by the local oxygen tension (Malladi et al., 2006; Fehrer et al., 2007; Buckley et al., 2010; Meyer et al., 2010; Sheehy et al., 2012; Pattappa et al., 2013), a detailed knowledge of how MSCs consume factors such as oxygen will play a key role in successfully engineering tissues of clinically relevant scales. Introducing gradients of regulatory factors such as oxygen into cell laden constructs, for example through the use of novel culture systems (Thorpe et al., 2013), may also be critical to engineering tissues with spatial complexity mimicking that observed in articular cartilage.

Chondrocytes are well adapted to low oxygen environments, deriving over 95% of their energy from glycolysis and demonstrating minimal oxygen consumption (Rajpurohit et al., 1996; Lee and Urban, 1997; Heywood and Lee, 2008). Undifferentiated MSCs possess a higher oxygen consumption rate than chondrocytes and derive a significantly larger portion of ATP generation via oxidative phosphorylation (Pattappa et al., 2011). Both osteogenic and chondrogenic differentiation of MSCs have been shown to alter the balance of oxidative phosphorylation and glycolysis, with culture of MSC pellets in chondrogenic media shown to result in a reduction in the oxygen consumption rate, and MSCs undergoing osteogenesis increasing their glucose consumption rate (Pattappa et al., 2011). In hypoxic conditions, MSCs have also been shown to derive a greater proportion of their ATP production from glycolysis (Pattappa et al., 2013).

In a tissue engineering context, while the external culture environment can be controlled with relative ease, depending on construct geometry, scale and cellular seeding density, large gradients in oxygen availability (and thus spatial gradients in cell viability and matrix deposition) can arise between the periphery and center of *in vitro* engineered grafts (Martin et al., 1999; Malda et al., 2004; Meyer et al., 2010; Buckley et al., 2011; Thorpe et al., 2013; Murphy et al., 2017; Westphal et al., 2017), potentially resulting in a necrotic core and graft failure. Understanding how parameters such as cell source, cell seeding density, oxygen consumption rate and culture environment influence oxygen gradients, and thus cellular metabolism and matrix synthesis, within engineered tissues will be critical to developing functional cartilage grafts of scale. The first objective of this study was to compare the oxygen consumption rate of chondrocytes and stem/progenitor cells isolated from bone marrow and infrapatellar fat pad following encapsulation into three dimensional hydrogels. Cell seeded constructs were maintained in chondrogenic medium supplemented with TGF-β3 for 24 days, with the oxygen levels measured within the engineered tissues at day 0 and day 24. The oxygen consumption rate was determined by fitting a computational model of oxygen transport and consumption within MSC laden hydrogels to experimental data obtained using implanted oxygen-sensitive micro-sensors. The second objective of this study was to explore how altering the external oxygen tension would affect local levels of oxygen availability and gradients within the engineered tissue. The final objective was to measure how oxygen levels and subsequent levels of matrix synthesis within engineered tissues depended on the initial cell seeding density.

## Materials and Methods

### Cell Isolation and Expansion

Bone marrow derived stem cells (BMSCs), infrapatellar fat pad derived stem cells (FPSCs) and chondrocytes (CCs) were isolated aseptically from the femoral diaphysis, the knee joint capsule and the femoral condyle, respectively, of 4 month old porcine donors as described previously (Buckley et al., 2010, 2011). Each group consisted of cells from a single donor (i.e., donors were not pooled). CCs were isolated from cartilage slices obtained from porcine femoral condyles via digestion with collagenase type II (0.5 mg/mL, Sigma–Aldrich, Dublin, Ireland) under constant rotation at 37°C until the tissue had visibly dissolved. The solution was filtered through a 40 μm nylon cell strainer (B.D. Falcon–Unitech) and centrifuged at 650 g for 10 min and the cells were then resuspended in expansion media consisting of high glucose Dulbecco’s modified Eagle’s medium (hgDMEM) GlutaMAX supplemented with 10% fetal bovine serum and 100 U/mL penicillin/streptomycin (all Gibco–Biosciences). Porcine infrapatellar fat pad harvested from the donor was washed with phosphate buffered saline (PBS), diced, and digested in the same manner as CCs. Bone marrow was aseptically harvested from the femoral diaphysis and the marrow was repeatedly aspirated by using a 16-gauge needle to break up large aggregates before centrifugation at 650 g for 5 min. The separated floating adipose layer was discarded, and the cell pellet was then resuspended in expansion media.

For cell counting, all cell suspensions (BMSCs, FPSCs and CCs) were triturated through 20-gauge needles to create single-cell suspensions and filtered through 40 μm nylon cell strainers. An aliquot of each suspension was treated with 4% acetic acid to lyse the red blood cells (BMSCs only) and viable mononuclear cells were counted by using a hemocytometer in the presence of 0.4% trypan blue. Mononuclear cells were initially seeded at a density of 5 × 10^3^ cells/cm^2^ (for FPSCs and CCs) or 50 × 10^3^ cells/cm^2^ (for BMSCs) in T-175 cm^2^ flasks (Sarstedt, Wexford, Ireland) and expanded in a 20% oxygen, 5% CO_2_ environment at 37°C. Following colony formation of BMSCs and FPSCs, cells were trypsinised, counted and expanded to passage 3 at a seeding density of 5 × 10^3^ cells/cm^2^ at each passage. CCs were expanded to passage 3 in the same manner. Complete media exchanges were performed twice weekly. We have previously demonstrated that porcine BMSCs isolated and expanded in this manner can differentiate down an osteogenic, adipogenic or chondrogenic pathway (Vinardell et al., 2011).

### Agarose Gel Constructs Fabrication and Culture

BMSC, FPSC and CC constructs were fabricated separately. Cell suspensions were mixed with 4% agarose (Type VII; Sigma-Aldrich) at a ratio of 1:1 at ∼40°C to yield a final gel concentration of 2% at a density of either 20 or 40 million cells/mL. The agarose/cell suspension was cast in stainless steel molds and allowed to set for 20 min to produce a solid slab, which was then cored with sterile biopsy punches to produce cylindrical constructs (Ø5 × 3 mm). Constructs were maintained for 24 days in a chemically defined chondrogenic medium consisting of high glucose DMEM supplemented with 100 U/mL penicillin/streptomycin (both from Gibco), 100 mg/mL sodium pyruvate, 40 mg/mL L-proline, 50 mg/mL L-ascorbic acid-2-phosphate, 1.5 mg/mL bovine serum albumin, 1 × insulin–transferrin–selenium, 100 nM dexamethasone (all Sigma-Aldrich), and 10 ng/mL of transforming growth factor-β3 (TGF-β3, ProSpec-Tany TechnoGene, Ltd.). Constructs were cultured in either a nominally 20 or 5% oxygen-controlled CO_2_ incubator (New Brunswick, Galaxy 48R). Each construct was cultured in an individual test dish (Figure 2) on an agarose bed to allow for greater, more homogenous oxygen distribution throughout the construct. Constructs were held firmly in position by the surrounding agarose bed to ensure that their position and orientation remained constant throughout the culture period. Complete media exchanges were performed twice weekly under atmospheric oxygen conditions using non-deoxygenated media.

### Experimental Design

**FIGURE 1:**
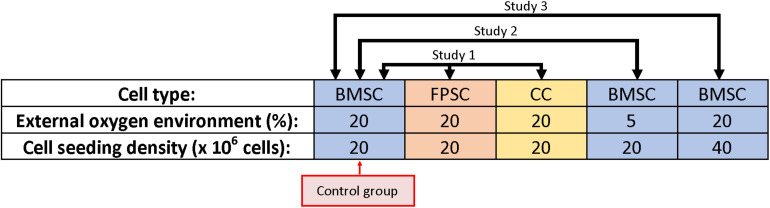
Study schematic; three studies were performed in parallel using a shared control group.

Three studies were performed in parallel using a shared control group (Figure 1):

**FIGURE 2:**
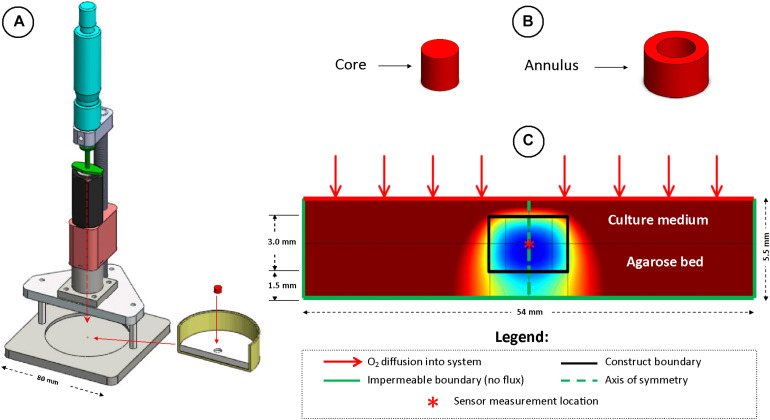
The local oxygen concentration in the center of *in vitro* engineered cartilage tissue grafts cultured in chondrogenic media was measured using a custom designed oxygen testing rig, test dish and **fiber** optic sensor **(A)**. Samples were separated into cores and annuli for spatial biochemical analysis **(B)**. Schematic of the finite element model developed to predict oxygen concentration gradients throughout constructs **(C)**.

1. Hydrogels were seeded with BMSCs, FPSCs or CCs at a cell density of 20 × 10^6^ cells/mL and cultured in 20% oxygen to investigate the influence of cell source on oxygen gradients and matrix deposition.
2. Hydrogels were seeded with BMSCs at a cell density of 20 × 10^6^ cells/mL and cultured in either 20 or 5% oxygen to investigate the influence of external oxygen environment.
3. Hydrogels were seeded with BMSCs at a cell density of either 20 × 10^6^ or 40 × 10^6^ cells/mL and cultured in 20% oxygen to investigate the influence of cell seeding density.

### Measurement of Local Oxygen Concentrations

A novel test rig (Figure 2A) was developed to allow accurate spatial positioning of sensor tips within the engineered constructs. Sample test dishes were prepared by pouring acellular 2% agarose into cell culture dishes (Iwaki, Ø60 mm), thus creating a 3 mm deep agarose bed. A recess (1.5 mm deep, Ø5 mm) was created in the center of the dish, into which samples to be tested were placed (thus leaving a 1.5 mm deep agarose bed underneath the sample). Sufficient medium was added to the dish resulting in a consistent depth of approximately 1 mm of medium above the construct.

Oxygen concentration at the center of each construct (O_2c_) was measured using a fiber optic oxygen transmitter (Microx TX; PreSens—Precision Sensing GmbH, Regensburg, Germany) and implantable, 140 μm tip diameter, fiber optic, oxygen micro-sensors. The sensors were calibrated using two-point calibration in oxygen free (nitrogen saturated and containing sodium sulphite) and air saturated medium at 37°C. At each time-point, a micrometer was used to accurately position the sensor tip at the geometrical center of the construct and the system was allowed to equilibrate for at least 3 h prior to each oxygen measurement.

### Prediction of Oxygen Concentration Within Agarose Constructs

Oxygen concentration in the constructs and throughout the culture dish was modeled using commercially available finite element modeling software (COMSOL Multiphysics 4.3) and is described by a diffusion-reaction type equation. The parameters used in the models are summarized in Table 1.

**TABLE 1:** Summary of parameters used for oxygen diffusion-reaction model.

Parameter	Symbol	Value	Units	Source
O_2_ diffusion coefficient in water	D_media_	3.0 × 10^–3^	mm^2^/s	Haselgrove et al., 1993; Lightfoot and Duca, 1999
O_2_ diffusion coefficient in 2% agarose	D_ag_	2.77 × 10^–3^	mm^2^/s	Gu et al., 2003; Sengers et al., 2005
Fluid phase volume fraction for 2% agarose	ϕ_f_	0.98		Calculated
Cell density at day 0 (low seeding density condition)	ρ	20 × 10^6^	cells/mL	Direct measurement (cell counting)
Cell density at day 0 (high seeding density condition)	ρ	40 × 10^6^	cells/mL	Direct measurement (cell counting)
O_2_ concentration at media surface (low O_2_ conditions)	C	50	μM	Direct measurement (oxygen sensor)
O_2_ concentration at media surface (high O_2_ conditions)	C	185	μM	Direct measurement (oxygen sensor)
O_2_ concentration at geometrical center of construct	O_2c_	measured	%	Direct measurement (oxygen microsensor)
Cell density at day 24 (core)	ρ_core_	measured	cells/mL	Direct measurement (DNA quantification)
Cell density at day 24 (annulus)	ρ_ann_	measured	cells/mL	Direct measurement (DNA quantification)
Michaelis—Menten constant	K_m_	63	μmol/mm^3^	Heywood et al., 2010
Cellular oxygen consumption rate	Q_m_	Unknown	mol/cell/s	Output predicted by numerical model

The reaction term followed Michaelis-Menten kinetics:

$$\frac{\partial c}{\partial t}=D\,\nabla^{2}c-\rho \,\frac{Q_{\text{m}}\,c}{K_{\text{m}}+c}$$

This equation describes a quasi-steady state reaction, which assumes that after an initial time period, the concentration of the reaction intermediates remains constant with time. Here, *c* is the oxygen concentration, *D* is the diffusion coefficient, *ρ* is the cell density and *K*_m_ is the oxygen concentration at which the reaction rate is at 50% of *Q*_m_. *Q*_m_ is defined as the maximum cellular oxygen consumption rate. However, the actual oxygen consumption rate of each cell is a function of the oxygen concentration at that particular location in accordance with Michaelis Menten kinetics. The value of *Q*_m_ was determined for each sample by using a linear regression approach (i.e., varying the value of *Q*_m_ that was input into the model until the output matched the experimentally observed O_2c_ value). Since oxygen concentration was measured at one location only, each *Q*_m_ value calculated was assumed to be representative of the maximum oxygen cellular consumption rate of each cell within the construct.

The diffusion coefficient in 2% agarose was determined using the Mackie and Meares relation:

$$\frac{D_{a\,g}}{D_{H_{2}\,O}}=\frac{\phi_{f}^{2}}{\left(2-\phi_{f}\right)}$$

so that *D*_*ag*_ = 2.77 × 10^–3^ mm^2^/s, where *ϕ_*f*_* (= 0.98) is the fluid phase volume fraction (Gu et al., 2003; Sengers et al., 2005). The Michaelis-Menten constant, *K*_m_ was set to 63 μmol/mm^3^ (Heywood et al., 2010). The construct was modeled as axisymmetric (Figure 2C). Oxygen diffusion through the culture media, *D*_*media*_ (= 3.0 × 10^–3^ mm^2^/s), was assumed to be the same as in water (Haselgrove et al., 1993; Lightfoot and Duca, 1999).

The oxygen concentration at the media surface was prescribed as 185 μM (measured using independently calibrated oxygen sensors) in the case of constructs cultured in the nominally 20% oxygen incubator and 50 μM for the constructs cultured in the 5% oxygen incubator. At the dish walls, base and at the axis of symmetry, the flux was set to zero. Day 0 time-point simulations were performed assuming uniform cell concentrations of ρ = 20 × 10^6^ or 40 × 10^6^ cells/mL throughout the construct. For day 24 simulations, however, the core and outer annular regions were modeled using separate ρ values derived from experimental data, to allow for discrepancies in cell proliferation/death between each region. The percentage change in cell concentration was calculated separately for each sample core and annulus by normalizing day 24 DNA values to mean day 0 DNA values. ρ values for day 24 models were then calculated by multiplying this ratio by the initial (day 0) seeding density, giving cell concentrations in each sample core (ρ_*core*_) and annulus (ρ_*ann*_), thereby accounting for spatial changes in cell number over the study duration. The cellular oxygen consumption rate *Q*_m_ was determined for each cell type and culture condition by systematically adjusting *Q*_m_ until the predicted O_2c_ matched the experimentally observed O_2c_. It should be noted that *Q*_m_ represents the mean oxygen consumption value for all cells in each construct and, in the case of an experimentally measured O_2c_ of 0%, the predicted value of *Q*_m_ is a minimum value of the oxygen consumption rate required to deplete oxygen to 0% at the center of the construct. Contour plots were then produced showing predicted oxygen gradients throughout the tissues. This modeling process was repeated for 3 different samples for each culture condition and time-point, using unique input parameters for *Q*_m_, *ρ*_*core*_, and *ρ*_*ann*_. Furthermore, frequency plots were also produced to demonstrate the volumetric fraction of each construct at specific oxygen levels.

### Biochemical Analysis

Each construct (*n* ≥ 3 per group) was separated into a core and annulus (to allow for spatial biochemical analyses (Figure 2B); using a 3 mm biopsy punch and their respective wet weights were measured. Each sample was then used for quantitative analysis of DNA, sulphated glycosaminoglycan (sGAG) and collagen content. For the former analyses, samples were digested with papain (125 μg/mL) in 0.1M sodium acetate, 5 mM L-cysteine-HCl, and 0.05M EDTA (pH 6.0, all Sigma-Aldrich) at 60°C under constant rotation for 18 h. DNA content was quantified using the Hoechst Bisbenzimide 33258 dye assay as described previously (Kim et al., 1988), with a calf thymus DNA standard. sGAG content was quantified using the dimethylmethylene blue dye-binding assay (DMMB; Blyscan, Biocolor Ltd., Northern Ireland) with a chondroitin sulfate standard. Collagen content was determined by measuring the hydroxyproline content. Samples were hydrolyzed at 110°C for 18 h in 38% HCl and assayed using a chloramine-T assay with a hydroxyproline : collagen ratio of 1:7.69 (Kafienah and Sims, 2004; Ignat’eva et al., 2007). Total sGAG and hydroxyproline values were normalized to DNA values.

### Histology and Immunohistochemistry

At selected time-points, constructs (*n* = 2) were fixed in 4% paraformaldehyde (Sigma-Aldrich) overnight at 4°C, rinsed with phosphate buffered saline (PBS), dehydrated and embedded in paraffin wax. Constructs were then cut in half and sectioned perpendicular to the disc face yielding 8 μm thick sections. Sections were stained with either 1% alcian blue 8GX (Sigma-Aldrich, Ireland) in 0.1M HCl for sGAG or picrosirius red for collagen. The deposition of collagen type II was identified through immunohistochemistry. Briefly, sections were rinsed with PBS and quenched of peroxidase activity for 20 min and treated with chondroitinase ABC (Sigma, 0.25 units/mL) in a humidified environment at 37°C for 1 h to enhance the permeability of the extracellular matrix by removal of chondroitin sulfate. Slides were again rinsed with PBS and blocked with 10% goat serum for 30 min. Sections were incubated with mouse monoclonal anti-collagen type II diluted 1:100 (Abcam, United Kingdom) (concentration 1 mg/mL) for 1 h at room temperature. After washing in PBS, the secondary antibody for type II collagen (Anti-Mouse IgG Biotin antibody produced in goat) (concentration 1 g/L) binding was applied for 1 h. Color was developed using the Vectastain ABC reagent (Vectastain ABC kit, Vector Laboratories, United Kingdom) for 45 min, followed by 5 min of exposure to peroxidase DAB substrate kit (Vector laboratories, United Kingdom). Slides were dehydrated through ethanol and xylene and mounted with Vectamount medium (Vector Laboratories, United Kingdom). Positive and negative controls (porcine cartilage and ligament) were included in the immunohistochemistry staining protocol for each batch.

### Statistical Analysis

All statistical analyses were performed using GraphPad Prism (Version 8.3) software. Results are reported as mean ± standard deviation. Groups were analyzed by a general linear model for analysis of variance. Tukey’s test was used to compare conditions. Significance was accepted at a level of *P* < 0.05 and population size n is stated where applicable.

## Results

### MSCs Adopt a Chondrocyte-Like Oxygen Consumption Rate Following Hydrogel Encapsulation and Stimulation With TGF-β3

**FIGURE 3:**
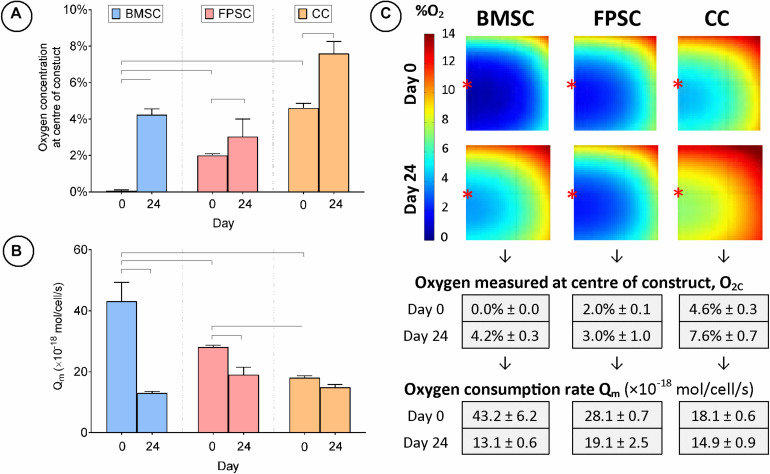
Cartilage tissue grafts engineered using various cell sources were cultured for 24 days. **(A)** Oxygen concentration at center of constructs. **(B)** Cellular oxygen consumption rate extrapolated from FE model. **(C)** FE contour plots showing predicted oxygen gradients and table of values from **(A,B)** (± SD). Significant differences between groups are indicated where *P* < 0.05 (*n* = 3). ^∗^Indicates the location of oxygen sensor measurement within the constructs.

Agarose hydrogels seeded with either BMSCs, FPSCs or CCs were cultured for 24 days in chondrogenic medium. The level of oxygen at the center of the construct (O_2c_) was measured at day 0 and 24 (Figures 3A,C). On day 0, the lowest O_2c_ levels were measured in BMSC seeded constructs (0.0% ± 0.0), whereas the highest levels were measured in the CC seeded constructs (4.6% ± 0.3). By day 24, O_2c_ levels had increased in BMSC constructs (4.2% ± 0.3) and were similar to those observed in FPSC seeded constructs (3.0% ± 1.0). The oxygen levels in CC constructs also increased over the duration of the study (from 4.6% ± 0.3 at day 0 to 7.6% ± 0.7 at day 24), whereas no significant change in oxygen levels were measured in FPSC constructs. Similar results were observed in a replicate study performed with cells isolated from a different donor (Supplementary Figure 1A).

While a likely explanation for the observed increases in oxygen levels within the constructs is a change in cellular metabolism as MSCs adopt a more chondrogenic phenotype with time in culture, an alternative explanation could be a change in total number of cells within the engineered tissue due to proliferation or cell death. To investigate this, average DNA content at day 24 was normalized to that of day 0 and the percentage change was calculated (Supplementary Figure 2A). Both BMSCs and FPSCs proliferated in both the annuli and the central core section of the samples over the study duration, with a ∼40% increase in DNA content. However, a reduction in CC cell numbers was observed, as evidenced by a ∼20% decrease in overall DNA content, with cell death being more prevalent in the higher oxygen annular section than in the core.

This experimental data on core oxygen levels and cell numbers was next used to develop finite element (FE) models of oxygen transport within the hydrogels and to predict cellular oxygen consumption rates. FE modeling of oxygen transport revealed that BMSCs were found to have highest cellular oxygen consumption rate at day 0 (Figure 3B), but this value dropped by approximately 70% (Q_m_ = 43.2 ± 6.2 vs. 13.1 ± 0.6 amol/cell/s) to a level similar to that of CCs by day 24. The CC oxygen consumption rate remained relatively unchanged throughout the study (Q_m_ = 18.1 ± 0.6 vs. 14.9 ± 0.9 amol/cell/s), suggesting that the observed change in oxygen concentration in CC constructs can be attributed to cell death (and hence a decrease in the number of oxygen consuming cells), as opposed to altered cell metabolism. Q_m_ values extrapolated for FPSCs show a significant decrease of approximately 30% in cellular oxygen consumption rate during the 24 days of culture (Q_m_ = 28.1 ± 0.7 vs. 19.1 ± 2.5 amol/cell/s). Despite this, oxygen gradients remained consistent throughout FPSC constructs over the duration of the study, as decreases in FPSC oxygen consumption rate were balanced with increases in cell numbers (Figure 3C).

**FIGURE 4:**
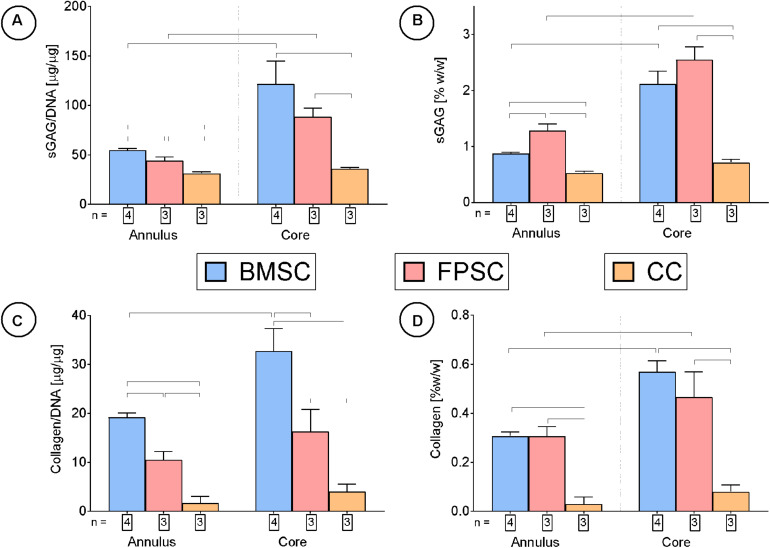
Cartilage tissue grafts engineered using various cell sources were cultured for for 24 days, then subsequently separated into cores and annuli and analyzed for DNA, sGAG and collagen content. Significant differences between groups are indicated where *P* < 0.05 (n shown on graph).

**FIGURE 5:**
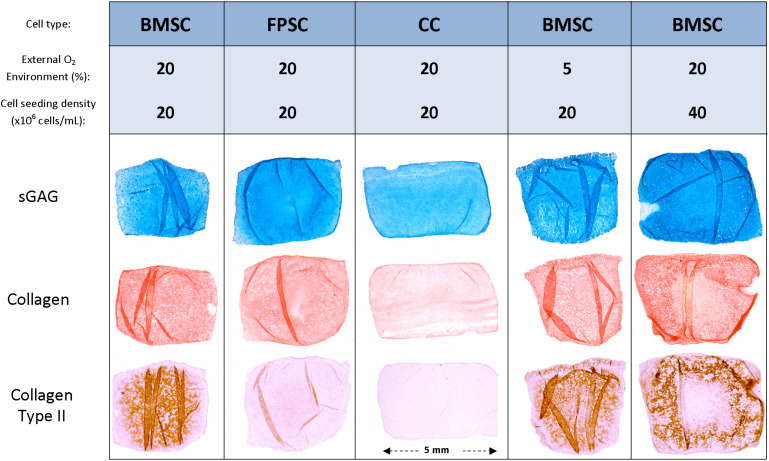
Histological and immuno-histochemical staining was performed on representative sections of cartilage tissue grafts engineered using various cell sources and cultured for 24 days.

After 24 days in culture, BMSCs had synthesized higher quantities of sGAG on a per-cell basis compared to FPSCs or CCs (Figure 4A). On a wet weight basis (%ww), greater levels of sGAG accumulation were observed in FPSC seeded constructs compared to both BMSC seeded constructs and CC seeded constructs (FPSC: 1.75 ± 0.08 %ww; BMSC: 1.29 ± 0.04 %ww; CC: 0.59 ± 0.01 %ww, for whole constructs). Spatial analysis of ECM deposition revealed higher levels of sGAG accumulation in the more hypoxic core than in the outer annulus of tissues engineered using stem cells (Figures 4A,B). Similar trends were observed for collagen deposition, with BMSCs synthesizing the most collagen when normalized to DNA content (Figure 4C), and with the greatest levels of accumulation also observed in the core (Figure 4D). On a percentage weight basis, comparable levels of total collagen accumulation were observed in FPSC and BMSC seeded constructs, which were higher than those observed in CC seeded constructs (FPSC: 0.36 ± 0.02 %ww; BMSC: 0.4 ± 0.02 %ww; CC: 0.04 ± 0.01 %ww, for whole constructs). Histological analysis revealed more intense staining for sGAG and collagen in BMSC and FPSC constructs when compared to CCs (Figure 5). Morphologically cells appeared similar in the central and peripheral regions of these engineered constructs, with a more well developed pericellular matrix observed in BMSC groups (Supplementary Figure 3). Only weak/negative staining for collagen type II was observed in FPSC and CC constructs, with BMSC constructs staining more strongly for type II collagen, particularly in the more hypoxic core region of the engineered tissue.

### Decreasing the External Oxygen Tension Reduces the Rate at Which BMSCs Consume Oxygen and Leads to Increased Matrix Accumulation

**FIGURE 6:**
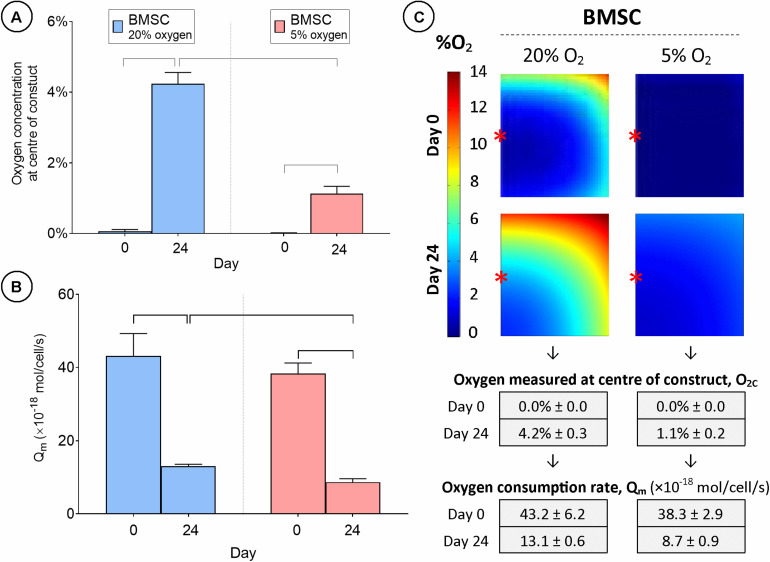
Cartilage tissue grafts engineered using bone marrow MSCs were cultured in either a 20% or low (5%) external oxygen environment for 24 days. **(A)** Oxygen concentration at center of constructs. **(B)** Cellular oxygen consumption rate extrapolated from FE model. **(C)** FE contour plots showing predicted oxygen gradients and table of values from **(A,B)** (± SD). Significant differences between groups are indicated where *P* < 0.05 (*n* = 3). ^∗^Indicates the location of oxygen sensor measurement within the constructs.

Although regions of low oxygen concentration correlated with increased ECM deposition in stem cell seeded constructs, it was unclear whether these low oxygen conditions were directly enhancing chondrogenesis, or if these regions of low oxygen within the engineered tissues were simply a function of the high levels of oxygen consumption by encapsulated cells. To address this question, BMSC-seeded constructs were cultured in either a 20 or 5% environment and local levels of oxygen availability in the engineered grafts were measured experimentally and oxygen consumption rates (Q_m_) were subsequently predicted using numerical models. O_2c_ was observed to be close to 0% for both external oxygen conditions at day 0 (Figures 6A,C). However, by day 24, local O_2c_ levels had increased significantly for both external oxygen environments (4.2% ± 0.3 for 20% oxygen and 1.1% ± 0.2 for 5% oxygen conditions).

Unsurprisingly, oxygen levels in the core of BMSC constructs cultured in a low (5%) oxygen environment were still lower than those in the core of constructs cultured in 20% oxygen conditions at day 24. While the boundary conditions of the FE model were set so that the O_2_ concentration at the air/media interface was equivalent to that of the air within the incubator, it was expected that O_2_ levels would drop across the 1 mm vertical diffusion distance between the media surface and the upper surface of the construct and that the maximum oxygen levels within the construct would be significantly lower than that of the air. FE simulations predicted higher concentrations of oxygen (ranging from 0 to 14% O_2_) throughout the constructs maintained in 20% oxygen compared to those maintained in low oxygen conditions, where the oxygen levels throughout much of the construct were less than 2% (Figure 6C and Supplementary Figure 4B). Q_m_ was shown to decrease in both cases over the culture period duration, but was significantly lower in the 5% oxygen (Q_m_ = 8.7 ± 0.7 amol/cell/s) condition than in 20% oxygen (Q_m_ = 13.1 ± 0.6 amol/cell/s) by day 24 (Figures 6B,C).

**FIGURE 7:**
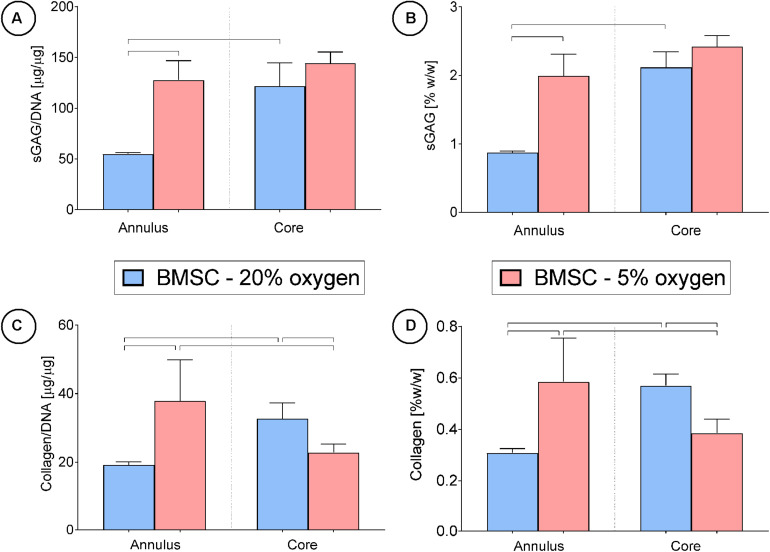
Cartilage tissue grafts engineered using bone marrow MSCs were cultured in either a 20% or low (5%) external oxygen environment for 24 days were separated into cores and annuli, then analyzed for DNA, sGAG and collagen content. Significant differences between groups are indicated where *P* < 0.05 (*n* = 4).

Although decreasing the external oxygen concentration to 5% had no effect on sGAG accumulation in the already low oxygen core, sGAG accumulation in the annulus of the construct maintained at an external oxygen concentration of 5% was approximately twice that measured in the annulus of the construct maintained at 20% oxygen (Figures 7A,B). Collagen deposition also increased in the annulus of the constructs maintained at 5% oxygen but decreased in the core (Figures 7C,D). The mean predicted oxygen level in the annulus was 9.0% for BMSC constructs maintained in 20% oxygen and 2.3% for those maintained in 5% oxygen, while the mean predicted oxygen level in the core was 6.4% for constructs cultured in 20% oxygen and 1.7% for those in 5% oxygen (see Supplementary Figure 4B for further details). This suggests that while environments of less than 2% oxygen concentration can continue to support sGAG synthesis, collagen synthesis can be negatively impacted. Positive alcian blue, picrosirius red and type II collagen staining was observed in all constructs (Figure 5), with the latter staining maximally in the core of constructs maintained at 20% oxygen.

### Increasing the Cell Seeding Density Lowers Oxygen Levels in Tissue Engineered Cartilage and Increases Extracellular Matrix Synthesis

**FIGURE 8:**
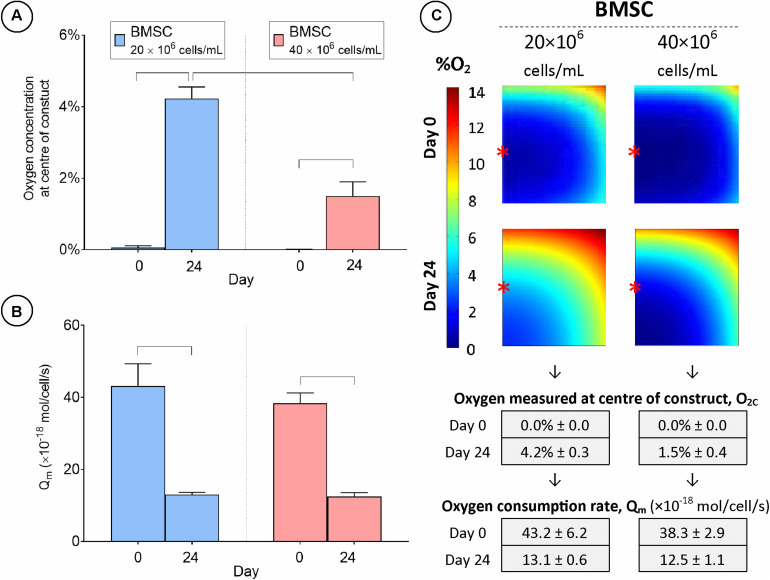
Cartilage tissue grafts engineered using bone marrow MSCs were seeded initially at a density of either 20 or 40 million cells/mL and cultured for 24 days. **(A)** Oxygen concentration at center of constructs. **(B)** Cellular oxygen consumption rate extrapolated from FE model. **(C)** FE contour plots showing predicted oxygen gradients and table of values from **(A,B)** (± SD). Significant differences between groups are indicated where *P* < 0.05 (*n* = 3). ^∗^Indicates the location of oxygen sensor measurement within the constructs.

To investigate the influence of cell seeding density on engineered cartilage tissue development, constructs were seeded at either 20 or 40 million cells/mL and cultured in chondrogenic medium for 24 days with the external oxygen concentration maintained at 20%. On day 0, O_2c_ was measured to be approximately 0% in both groups (Figure 8A). This value increased for both conditions over the study period despite significant cell proliferation (Supplementary Figure 2C) but as expected was significantly greater in the lower seeding density group by day 24 (4.2% ± 0.3 vs. 1.5% ± 0.4 O_2c_). A higher cell seeding density was predicted to lead to the development of a much larger hypoxic core region at both day 0 and 24 (Figure 8C), although the size of this region was not as large as that for the lower cell seeding density when maintained at 5% external oxygen concentration (compare Figures 6C, 8C). Extrapolated values for the average cellular oxygen consumption rate at day 24 were similar for both cell seeding densities, but significantly lower than day 0 values (Figure 8B).

**FIGURE 9:**
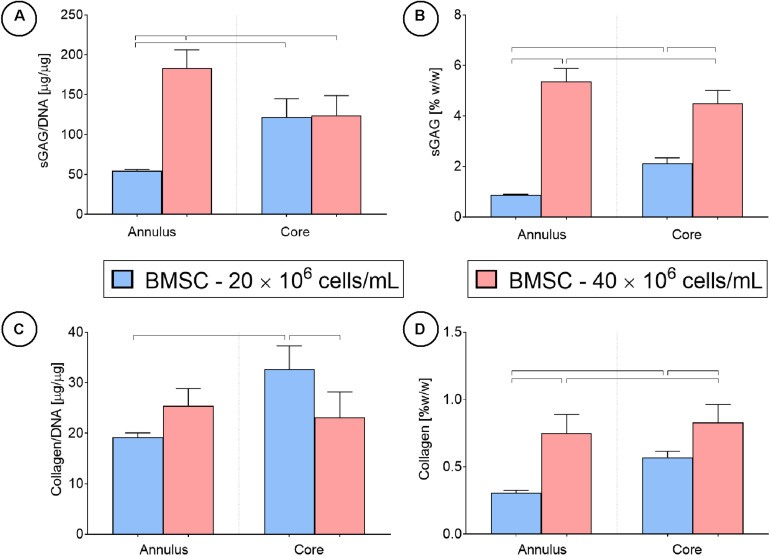
Cartilage tissue grafts engineered using bone marrow MSCs were seeded initially at a density of either 20 or 40 million cells/mL for 24 days, then separated into cores and annuli and analyzed for DNA, sGAG and collagen content. Significant differences between groups are indicated where *P* < 0.05 (*n* = 4).

Spatial biochemical analysis revealed that doubling the initial seeding density resulted in a large increase in sGAG synthesis (on a per cell basis) and accumulation in the construct annulus, as measured by increases in sGAG/DNA (×3.5 times, Figure 9A) and sGAG as a % of wet weight (×6.7 times, Figure 9B). Increasing the seeding density also led to higher sGAG accumulation in the core region, but not when sGAG accumulation was normalized to DNA content. Collagen synthesis was also enhanced in the annulus with increasing seeding density but was reduced in the core region (Figures 9C,D). More intense staining for sGAG was observed throughout the high seeding density constructs (Figure 5), while collagen staining was located primarily in the annular region. Immuno-histochemical staining for collagen type II clearly showed the effects of varying the seeding density, with collagen II concentrated in the core of the low seeding density constructs and around the periphery of the high seeding density constructs.

## Discussion

It has previously been demonstrated that the external oxygen concentration can influence MSC metabolism, differentiation and matrix synthesis (Fehrer et al., 2007; Buckley et al., 2010; Meyer et al., 2010; Pattappa et al., 2013). In the current study, a combined experimental and numerical approach was utilized to characterize the spatial oxygen environment within cartilage tissues engineered using various sources of cells. Oxygen levels measured within tissues seeded with BMSCs and CCs increased significantly over the study duration. However, biochemical analysis and finite element modeling revealed different causes for these increases: MSCs were found to possess a high initial oxygen uptake rate, but to adopt a more chondrocyte-like oxygen consumption profile following chondrogenic differentiation, whereas increases in oxygen concentration within CC constructs were attributed to decreases in cell number. Spatial biochemical analysis of the engineered tissues revealed a correlation between low oxygen regions and enhanced levels of cartilage specific extra cellular matrix deposition.

Direct measurements of oxygen levels within the constructs provided an insight into the local cellular environment during chondrogenesis in 3D hydrogels. BMSCs and FPSCs were found to have proliferated significantly over the study duration, while the CC population reduced in number. Using this data, the average oxygen consumption rate of BMSCs was estimated to be 43.2 amol/cell/s prior to chondrogenic differentiation, which is comparable to values previously reported for BMSCs of 33 amol/cell/s (Pattappa et al., 2013). FPSCs were estimated to utilize oxygen at a rate of 28.1 amol/cell/s at day 0. CC oxygen consumption did not change significantly throughout the study, with an average rate of 16.5 amol/cell/s, which is reasonably consistent with previously reported values of 19.4 amol/cell/s (Heywood and Lee, 2010). However, by day 24, both BMSCs and FPSCs had adopted a similar oxygen uptake (13.1 and 19.1 amol/cell/s, respectively) to that of CCs. In fact, it appears that this switch occurred prior to day 14 (see Supplementary Figure 5), but continuous monitoring of oxygen levels within the construct and an increased number of FE models and analyses would be required to pinpoint when this switch occurs. During monolayer expansion, MSCs are known to possess the ability to adapt their oxygen consumption to changes in the oxygen environment (Pattappa et al., 2013). The apparent switch to a more glycolytic metabolism reported in the current study has also been observed previously in MSC pellets (Pattappa et al., 2011), whereas increases in oxidative metabolism has been associated with a loss of chondrocyte phenotype (Heywood and Lee, 2008).

In the current study, it was demonstrated that BMSCs possess a higher initial oxygen uptake rate than FPSCs and this resulted in an O_2C_ measurement of 0% for all BMSC-laden constructs at day 0. A possible explanation for this is that these cells reside within different oxygenated niches *in vivo* and have adapted metabolisms to reflect this. Despite this, both groups were successfully induced to adopt a more chondrogenic oxygen consumption rate and synthesize cartilage-specific extracellular matrix. Consistent with previously reported findings (Meyer et al., 2010; Buckley et al., 2011), matrix deposition was generally maximal in regions of low oxygen. Even when cultured at 20% oxygen, a hypoxic core region developed in MSC laden hydrogels where matrix deposition was greatest. Culturing in a low oxygen environment effectively expanded this hypoxic region to include the entirety of the construct, thereby enhancing matrix synthesis within the annular region also. However, this also had the effect of further reducing oxygen levels in the core from an average concentration (by volume) of 6.4–1.7% oxygen, which, while having no net effect on sGAG content, appeared to inhibit collagen synthesis. This suggests that, while both sGAG and collagen synthesis is enhanced by culture in 5% oxygen, very low oxygen environments can be detrimental to collagen production. However, since MSCs have been observed to increase their glucose uptake when maintained in hypoxia (Pattappa et al., 2013; Naqvi and Buckley, 2015), the lower oxygen environment predicted throughout the entirety of the constructs cultured in 5% oxygen will also likely lead to a depletion of glucose in the core region, thus potentially inhibiting collagen synthesis. Indeed, it has been reported that, while collagen deposition by MSCs remains unaffected in either low oxygen or low glucose environments, hypoxia combined with deprivation of glucose leads to significantly reduced collagen synthesis, whereas sGAG synthesis is enhanced under these conditions, when compared to those cultured in 20% oxygen (Naqvi and Buckley, 2015). Follow-up studies measuring glucose consumption in engineered tissues are required to further test this hypothesis.

Both FPSCs and BMSCs secreted higher levels of sGAG and collagen compared to CCs. While we have previously observed that FPSCs and BMSCs secrete higher levels of cartilage matrix specific extracellular matrix components *in vitro* (Sheehy et al., 2011; Vinardell et al., 2012), others have reported superior chondrogenesis with CCs compared to BMSCs following hydrogel encapsulation (Mauck et al., 2006; Farrell et al., 2012). It should be noted that the CCs used in this study were first expanded on tissue culture plastic prior to hydrogel encapsulation, which is known to result in dedifferentiation of the cells. This is similar to what occurs with clinical cell-based therapies such as autologous chondrocyte implantation. This likely contributes to the relatively lower levels of chondrogenesis observed with the CCs compared to the other cell types. Further studies are required to assess how such monolayer expansion influences the metabolic phenotype of CCs.

Increasing the cell seeding density also had the effect of expanding the hypoxic core region (Figure 8C) and again enhancing matrix accumulation in the annulus, at the cost of reduced collagen synthesis (on a per cell basis) in the core. As discussed previously, this may be due to low oxygen conditions and/or glucose depletion in constructs seeded at high densities. However, as native cartilage consists of a zonal architecture, with gradients in biochemical content and functional properties throughout the tissue (Chen et al., 2001; Julkunen et al., 2009; Gannon et al., 2012), introducing nutrient gradients, and even selective localized scarcity in nutrient availability, may be ultimately desirable when attempting to recapitulate native-like tissue composition and architecture.

This paper presents a novel approach to measuring and modeling the oxygen consumption rates of stem cells within engineered tissues, although it does involve making certain assumptions. While cell density was estimated separately for each core and annulus region, cell density gradients, which are not fully accounted for, will likely develop within these regions. Relying solely on a DNA assay to measure changes in cell number within the hydrogels is also a limitation; DNA of dead cells may remain trapped within the hydrogel and be detected by the biochemical assay. Future studies should look to combine such assays with imaging to assess spatial changes in cell proliferation and death. Also, the *K*_m_ value used for all cell types was a value reported for bovine articular chondrocytes and was assumed not to vary throughout the study. Therefore, it is proposed that future studies should involve accurately measuring cell density gradients, quantifying temporal changes in *K*_m_ for each culture condition, monitoring both oxygen and glucose concentrations more frequently (particularly in the early phase to monitor the onset of differentiation) and at multiple locations throughout the tissues and the development of dual-species oxygen/glucose diffusion-reaction numerical models. Other improvements to the model would include altering the diffusion rate of different species through the hydrogel as a function of matrix deposition by cells, locations and considering factors such as acidification and changing lactate concentrations. Furthermore, exploring the donor-to-donor variability in oxygen consumption rates should be more robustly addressed in future studies.

The results of this study can also inform the future clinical development of cell and tissue engineering based strategies for articular cartilage regeneration. By understanding, monitoring and modeling how oxygen consumption rates change with time in culture, in the future it should be possible to use such data as a metric of MSC differentiation and in the design of culture environments that temporally modulate external oxygen levels to accelerate chondrogenesis and the functional development of tissue engineered cartilage grafts. For allogenic tissue engineering strategies, this might be achieved by first carefully characterizing the oxygen consumption rates of a specific lot of donor MSCs as they undergo chondrogenesis, and then implementing donor-specific culture conditions when scaling-up the engineering of grafts using banks of these donor cells. For autologous tissue engineering strategies, it will likely require the on-line monitoring of oxygen consumption within each culture, with real-time control of the external oxygen conditions to provide the ideal conditions for these patient-specific grafts.

## Conclusion

Understanding how the local cellular environment influences cell metabolism, phenotype and matrix synthesis will be crucial to engineering functional cartilage tissue grafts of a clinically relevant scale. Oxygen and nutrient gradients and consumption rates within tissue grafts can be influenced by varying factors such as the external culture environment oxygen tension, cell seeding density and construct scale. However, these gradients will be often in a state of flux, varying both spatially and temporally, where the local environment will influence cell metabolism, which in turn will influence the cellular environment. These studies present a combined experimental and numerical approach to characterizing these gradients and cellular responses and, potentially provides a framework which in the future may allow tissue engineers to control these gradients to engineer native-like articular cartilage. It was demonstrated that MSCs undergoing chondrogenesis adapt their oxygen consumption profile to be more similar to that of chondrocytes. Therefore, determining if MSCs used to engineer cartilage grafts adopt an oxygen consumption profile similar to chondrocytes might be considered a key determinant of the success of stem cell-based cartilage tissue engineering strategies.

## Data Availability Statement

The raw data supporting the conclusions of this article will be made available by the authors, without undue reservation.

## Author Contributions

SC performed all the experimental activities and sample analyses. All authors were involved in study design, interpretation and presentation of data and preparation of this article.

## Conflict of Interest

The authors declare that the research was conducted in the absence of any commercial or financial relationships that could be construed as a potential conflict of interest.

## Supplementary Material

Supplementary Figure 1 A replicate study was performed using cells from a separate set of donors and oxygen concentration in the center of each construct was measured.

Supplementary Figure 2 The percentage change in cell density in sample cores and annuli following 24 days of culture.

Supplementary Figure 3 High-magnification (20x) images of representative sections stained with Alcian Blue and Nuclear Fast Red following 24 days of culture.

Supplementary Figure 4 FEA models were analyzed to determine the percentage volume of each sample that contained nodes with O_2_ values that lay within the specified oxygen ranges at day 24.

Supplementary Figure 5 Oxygen concentration as measured in the center of constructs from each study group for both the main and replicate studies combined.
